# Application of machine learning algorithm in prediction of lymph node metastasis in patients with intermediate and high-risk prostate cancer

**DOI:** 10.1007/s00432-023-04816-w

**Published:** 2023-05-02

**Authors:** Xiangrong Wang, Xiangxiang Zhang, Hengping Li, Mao Zhang, Yang Liu, Xuanpeng Li

**Affiliations:** Department of Urology, Gansu Provincial Hospital, Lanzhou, Gansu China

**Keywords:** Intermediate- and high-risk prostate cancer, Lymph node metastasis, Seer, Machine learning, Calculator

## Abstract

**Purpose:**

This study aims to establish the best prediction model of lymph node metastasis (LNM) in patients with intermediate- and high-risk prostate cancer (PCa) through machine learning (ML), and provide the guideline of accurate clinical diagnosis and precise treatment for clinicals.

**Methods:**

A total of 24,470 patients with intermediate- and high-risk PCa were included in this study. Multivariate logistic regression model was used to screen the independent risk factors of LNM. At the same time, six algorithms, namely random forest (RF), naive Bayesian classifier (NBC), xgboost (XGB), gradient boosting machine (GBM), logistic regression (LR) and decision tree (DT) are used to establish risk prediction models. Based on the best prediction performance of ML algorithm, a prediction model is established, and the performance of the model is evaluated from three aspects: area under curve (AUC), sensitivity and specificity.

**Results:**

In multivariate logistic regression analysis, T stage, PSA, Gleason score and bone metastasis were independent predictors of LNM in patients with intermediate- and high-risk PCa. By comprehensively comparing the prediction model performance of training set and test set, GBM model has the best prediction performance (F1 score = 0.838, AUROC = 0.804). Finally, we developed a preliminary calculator model that can quickly and accurately calculate the regional LNM in patients with intermediate- and high-risk PCa.

**Conclusion:**

T stage, PSA, Gleason and bone metastasis were independent risk factors for predicting LNM in patients with intermediate- and high-risk PCa. The prediction model established in this study performs well; however, the GBM model is the best one.

## Introduction

According to the global cancer statistics in 2020, PCa ranks sixth in incidence rate and seventh in mortality in China. (Cao 2020). Pelvic lymph node metastasis (PLNM) accounts for about 15% of all newly diagnosed PCa patients, which is related to biochemical recurrence (BCR) and distant metastasis (DM) after treatment (von Bodman et al. 2010; Wilczak et al. 2018). Gervasi et al. reported that the 10-year risk of DM in lymph node positive patients was 83%, and the 10 year risk of death from PCa was 57% (Wagner et al. 2008). Extended pelvic lymph node dissection (ePLND) has become an integral part of radical prostatectomy (RP), while the American Association of Urology (AUA) and the European Association of Urology (EAU) recommend that low-risk patients do not need ePLND; ePLND is an option for patients with intermediate- and high-risk PCa whose Briganti nomogram predicts that the probability of LNM is greater than 5% (Engel et al. 2010; Lestingi et al. 2021). Therefore, the clinical staging of PCa is the key to precision medicine, and accurate identification of PLNM of PCa patients is crucial to determine the appropriate treatment plan (Hou et al. 2021; Mottet et al. 2017).

At present, many studies have reported that non-invasive imaging techniques can be used to predict LNM of PCa before treatment. CT and MRI, the most commonly used in clinic, can assess the status of pelvic lymph nodes by examining their size. Both of them have no obvious advantages and disadvantages, with a sensitivity of about 40% and a specificity of about 82% (Créhange et al. 2012; Hövels et al. 2008). Von Below et al. showed that multi parameter MRI (mpMRI) is more sensitive and specific than MRI in detecting tumors and lymph nodes, but it is easy to lose signal or image distortion in DWI sequence (von Below et al. 2016). Similarly, PSMA PET/CT has been widely used to detect PCa in prostate, soft tissue and bone, however, and its detection rate of 2–5 mm lymph node invasion is about 60% (Hofman et al. 2018; van Leeuwen et al. 2017). In addition, new imaging technologies are being developed such as MR lymphography with superparamagnetic iron oxide (SPIO) nanoparticles and targeted positron emission tomography imaging (PET) (Muteganya et al. 2018). Their efficacy of prediction for the NLM is still unclear.

Recently, scientists have made great efforts to explore different methods for more accurately evaluating the risks of LNM. However, due to the complexity of medical data, there are important connections between various factors, and certain differences in the calculation methods of models. Therefore, machine learning (ML) has become a powerful tool for improvement of clinical strategies in the field of medical research (Mirza et al. 2019; Oliveira 2019). Compared with traditional regression analysis, ML algorithm has significant advantages in prediction performance in large databases (Bi et al. 2019; Wang et al. 2020). Tian et al. established RDA model using ML to accurately predict LNM of early gastric cancer (Tian et al. 2021). Li et al. established XGB model to predict LNM of patients with osteosarcoma (Li and Liu et al. 2022). Li et al. established RF model to better predict LNM of Ewing’s sarcoma (Li and Zhou et al. 2022).

To our knowledge, there is no effective ML model for predicting risks of LNM of PCa. Therefore, in this study, we established a new model for predicting risks of LNM in patients with intermediate- and high-risk PCa through 6 ML methods based on the clinical and histopathological parameters that are closely related to the prognosis of the PCa in the SEER database.

## Materials and methods

### Study population

**Fig. 1:**
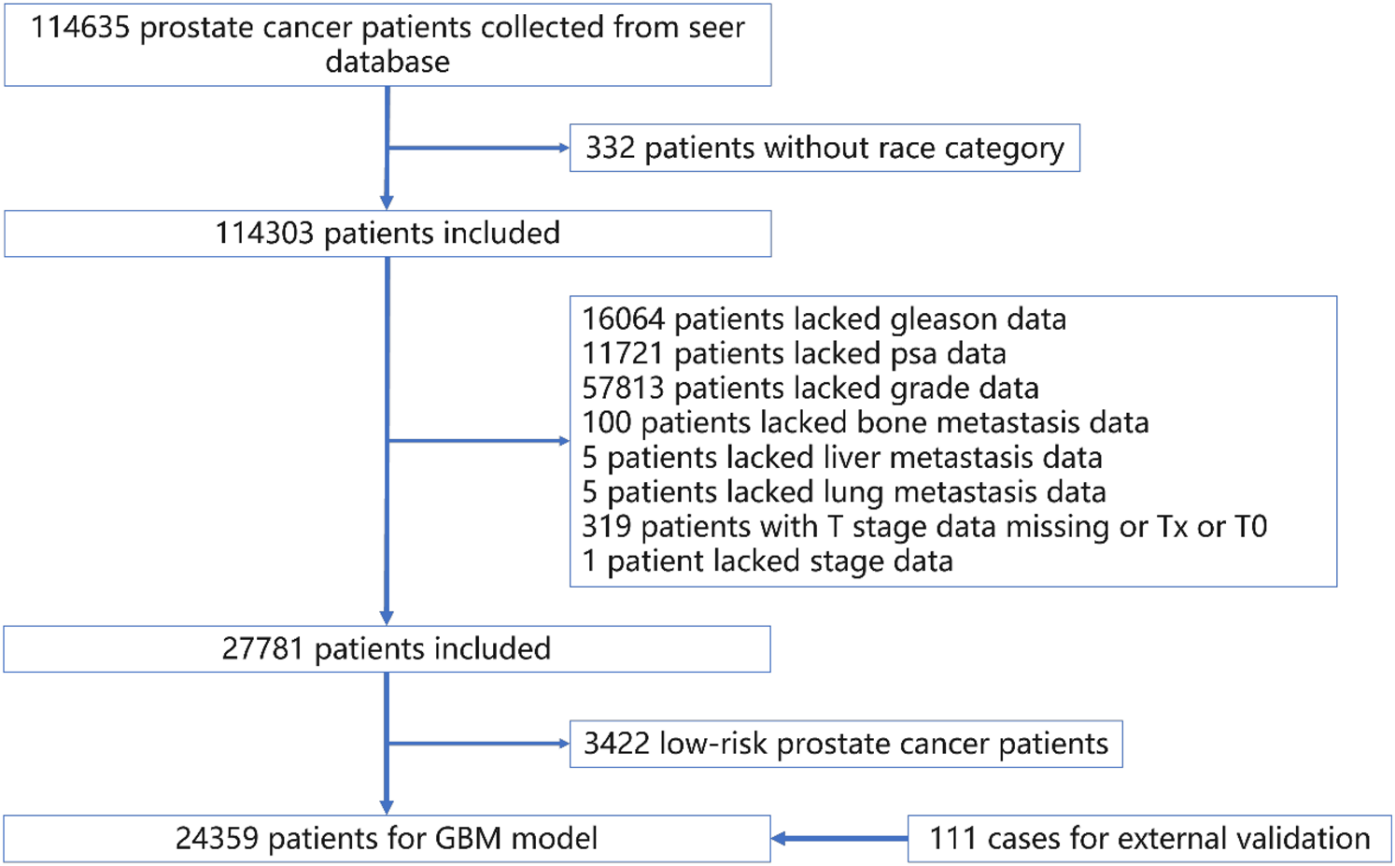
Model development process workflow

The training set and test set were recruited from the SEER database for patients diagnosed with intermediate- and high-risk PCa from 2000 to 2019. The patients diagnosed as intermediate- and high-risk PCa by Gansu Provincial Hospital from 2012 to 2018 will be taken as the validation set. Inclusion criteria were as follows: (1) patients with primary prostate cancer confirmed by the case; (2) at least meet one of PSA ≥ 10 ng/ml, Gleason score ≥ 7 or T stage ≥ T2b; (3) The clinical and pathological data and survival period were complete. Exclusion criteria: (1) no complete clinicopathological data and survival period; (2) PSA < 10 ng/ml, Gleason score < 7 and T1–T2a. Since the study was retrospective and the data were from an open database, informed consent was not used. The detailed screening process is shown in Fig. 1.

### Establishment of predictive model

In this study, we compared the pathological characteristics selected from SEER database and external validation set, and analyzed the risk factors for predicting LNM using single factor analysis. Multivariate logistic regression analysis was used to evaluate the variables, and independent predictors related to LNM were obtained. Then we selected 6 common prediction models based on ML to predict LNM of intermediate- and high-risk PCa. We have established six models: random forest (RF), naive Bayesian classifier (NBC), xgboost (XGB), gradient boosting machine (GBM), logistic registration (LR) and decision tree (DT). The SEER dataset was divided by a ratio of 70:30. 70% is used for machine algorithm training, 30% is used for testing, and external verification was used as a separate verification set. In the training process of ML algorithm, each model is cross verified for 10 times to maintain the stability of the model, and the best super parameters are selected using random search method. The F1 score, AUROC, sensitivity and specificity of each model are comprehensively evaluated, compared the performance differences of different models, and selected the model with the highest accuracy as the final model according to the comprehensive score. Finally, the accuracy and generalization of the selected best prediction model are further verified using an independent external verification set.

### Assessment of prediction model

We used area under curve (AUC) to evaluate the accuracy of each model. Considering the possibility of over fitting or under fitting, we combined the sensitivity and specificity of each model to obtain F1 score. In addition, we use decision curve analysis to test the prediction accuracy of the model.

### Statistical analysis

We used SEER * STAT statistical software to extract training sets and test sets from SEER database. Hospital patients as an external validation set. All patient data were analyzed with SPSS V.25.0. Continuous variables are represented by the median of interquartile interval (IQR), and categorical variables are represented by values and proportions. Wilcoxon rank sum test is used for continuous variables, and chi square test or Fisher exact test is used for categorical variables. Univariate and multivariate logistic regression were used to analyze the risk factors of lymph node metastasis in high-risk PCa. *P* values lower than 0.05 were statistically significant. Adjusted odds ratios (ORs) and corresponding 95% confidence intervals (95% CI) were calculated. The modeling process is implemented through the Sci Kit Learn library (version 0.19.2) in Python (version 3.7.1). Test the training set with RF, NBC, XGB, GBM, LR and DT, and establish a prediction model. The relative importance of each input variable in each model is analyzed. We used 10 times cross validation and ROC curve analysis on the training set to test the performance of the model. Finally, the prediction accuracy of GBM model is further verified by decision curve analysis.

## Results

### Baseline characteristics

A total of 24,470 patients with intermediate- and high-risk PCa were included in this study, including 24,359 from SEER database and 111 from our hospital’s external validation set. Patients were divided into two groups according to whether they had LNM. There were significant differences between the two groups (patients with or without LNM) in terms of grade (*p* < 0.001), T stage (*p* < 0.001), M stage (*p* < 0.001), Stage (*p* < 0.001), Gleason (*p* < 0.001), PSA (*p* < 0.001), bone metastasis (*p* < 0.001), liver metastasis and lung metastasis (*p* < 0.001) (Table 1).

**Table 1:** Describe the study population according to whether there is lymph node metastasis

Variable	Level	Overall (*N* = 24,470)	Lymph node metastases		*P*
			No(*N* = 22,688)	Yes(*N* = 1782)	
Category [*n* (%)]	single center	111 (0.5)	104 (0.5)	7 (0.4)	0.692
	seer	24,359 (99.5)	22,584 (99.5)	1775 (99.6)	
Age [median (IQR)]	NA	64 (59,69)	65 (57,71)	64 (59,69)	0.186
Race [*n* (%)]	American Indian/Alaska Native	126 (0.5)	113 (0.5)	13 (0.7)	0.043*
	Asian or Pacific Islander	1547 (6.3)	1458 (6.4)	89 (5.0)	
	Black	3385 (13.8)	3123 (13.8)	262 (14.7)	
	White	19,412 (79.3)	17,994 (79.3)	1418 (79.6)	
Grade [*n* (%)]	1	664 (2.7)	659 (2.9)	5 (0.3)	< 0.001*
	2	9675 (39.5)	9500 (41.9)	175 (9.8)	
	3	6990 (28.6)	6539 (28.8)	451 (25.3)	
	4	3317 (13.6)	3041 (13.4)	276 (15.5)	
	5	3824 (15.6)	2949 (13.0)	875 (49.1)	
T [*n* (%)]	T1	974 (4.0)	942 (4.2)	32 (1.8)	< 0.001*
	T2	13,085 (53.5)	12,888 (56.8)	197 (11.1)	
	T3	9974 (40.8)	8547 (37.7)	1427 (0.8)	
	T4	437 (1.8)	311 (1.4)	126 (7.1)	
M [*n* (%)]	M0	24,221 (99.0)	22,559 (99.4)	1662 (93.3)	< 0.001*
	M1	249 (1.0)	129 (0.6)	120 (6.7)	
Stage [*n* (%)]	I	118 (0.5)	118 (0.5)	0 (0)	< 0.001*
	II	11,957 (48.9)	11,957 (52.7)	0 (0)	
	III	10,484 (42.8)	10,484 (46.2)	0 (0)	
	IV	1911 (7.8)	129 (0.6)	1782 (1)	
Gleason [*n* (%)]	≤ 6	1727 (7.1)	1699 (7.5)	28 (1.6)	< 0.001*
	7	16,028 (65.5)	15,333 (67.6)	695 (39.0)	
	8	3939 (16.1)	3531 (15.6)	408 (22.9)	
	9	2579 (10.5)	2000 (8.8)	579 (32.5)	
	10	197 (0.8)	125 (0.6)	72 (4.0)	
PSA [median (IQR)]	NA	7.2(5.3,11.3)	7(5.2,10.8)	11.5(7,23)	< 0.001*
Bone metastasis [*n* (%)]	M0	24,283 (99.2)	22,579 (99.5)	1704 (95.6)	< 0.001*
	M1	187 (0.8)	109 (0.5)	78 (4.4)	
Liver metastasis [*n* (%)]	M0	24,462 (99.9)	22,687 (99.9)	1775 (99.6)	< 0.001*
	M1	8 (0.1)	1 (0.1)	7 (0.4)	
Lung metastasis [*n* (%)]	M0	24,458 (99.9)	22,682 (99.9)	1776 (99.7)	< 0.001*
	M1	12 (0.1)	6 (0.1)	6 (0.3)	
Time [median (IQR)]	NA	11(5,17)	11(4,17)	11(5,17)	0.83

### Univariate and multivariate analyses of potential factors for predicting lymph node metastases

In univariate analysis, race (*p* = 0.049), grade (*p* < 0.001), T (*p* < 0.001), M (*p* < 0.001), stage (*p* < 0.001), Gleason score (*p* < 0.001), PSA (*p* < 0.001), bone metastasis (*p* < 0.001), liver metastasis (*p* < 0.001), and lung metastasis (*p* < 0.001) were significantly related to the occurrence of lymph node metastasis of intermediate- and high-risk PCa. There was no significant difference in age between the two groups. Multivariate logistic regression analysis showed that T (*p* = 0.016), Gleason (*p* = 0.031), PSA (*p* = 0.033) and bone metastasis (*p* < 0.001) were independent predictors of LNM (Table 2).

**Table 2:** Single- and multi-factor logistic regression analysis for the modeling group

Variable	Univariate analysis		Multivariate analysis	
	OR (95% CI)	*p* value	OR (95% CI)	*p* value
Age(years)		0.486	0.989(0.946–1.035)	0.638
Age(N0)	63.610(63.515–63.701)			
Age(N1)	63.731(63.399–64.057)			
Race		0.049*	1.080(0.657–1.774)	0.762
American Indian/Alaska Native	0.103(0.049–0.157)			
Asian or Pacific Islander	0.058(0.046–0.069)			
Black	0.077(0.068–0.086)			
White	0.073(0.069–0.077)			
Grade		< 0.001*	0.806(0.504–1.288)	0.367
1	0.008(0.001–0.014)			
2	0.018(0.015–0.021)			
3	0.065(0.059–0.070)			
4	0.083(0.074–0.093)			
5	0.229(0.216–0.242)			
T		< 0.001*	1.686(1.101–2.581)	0.016*
T1	0.033(0.022–0.044)			
T2	0.015(0.013–0.017)			
T3	0.143(0.136–0.150)			
T4	0.287(0.245–0.330)			
M		< 0.001*	0.002(0.001–0.003)	0.954
M0	0.069(0.066–0.072)			
M1	0.484(0.421–0.546)			
Stage		< 0.001*	0.121(0.004–0.174)	0.901
I	0.002(0.001–0.003)			
II	0.325(0.021–0.476)			
III	0.375(0.234–0.442)			
IV	0.249(0.069–0.076)			
Gleason		< 0.001*	1.709(1.050–2.784)	0.031*
≤ 6	0.016(0.010–0.022)			
7	0.043(0.040–0.046)			
8	0.104(0.094–0.114)			
9	0.225(0.209–0.241)			
10	0.362(0.294–0.430)			
PSA		< 0.001*	1.019(1.002–1.037)	0.033*
Psa(N0)	9.670(9.553–9.787)			
Psa(N1)	17.424(16.607–18.241)			
Bone metastasis		< 0.001*	0.202(0.094–0.436)	< 0.001*
M0	0.070(0.067–0.074)			
M1	0.416(0.345–0.488)			
Liver metastasis		< 0.001*	5.446(0.350–9.810)	0.226
M0	0.073(0.069–0.076)			
M1	0.875(0.579–1.171)			
Lung metastasis		< 0.001*	0.248(0.031–1.958)	0.186
M0	0.073(0.069–0.076)			
M1	0.500(0.168–0.832)			

### Screening and validation of the best machine learning model

**Fig. 2:**
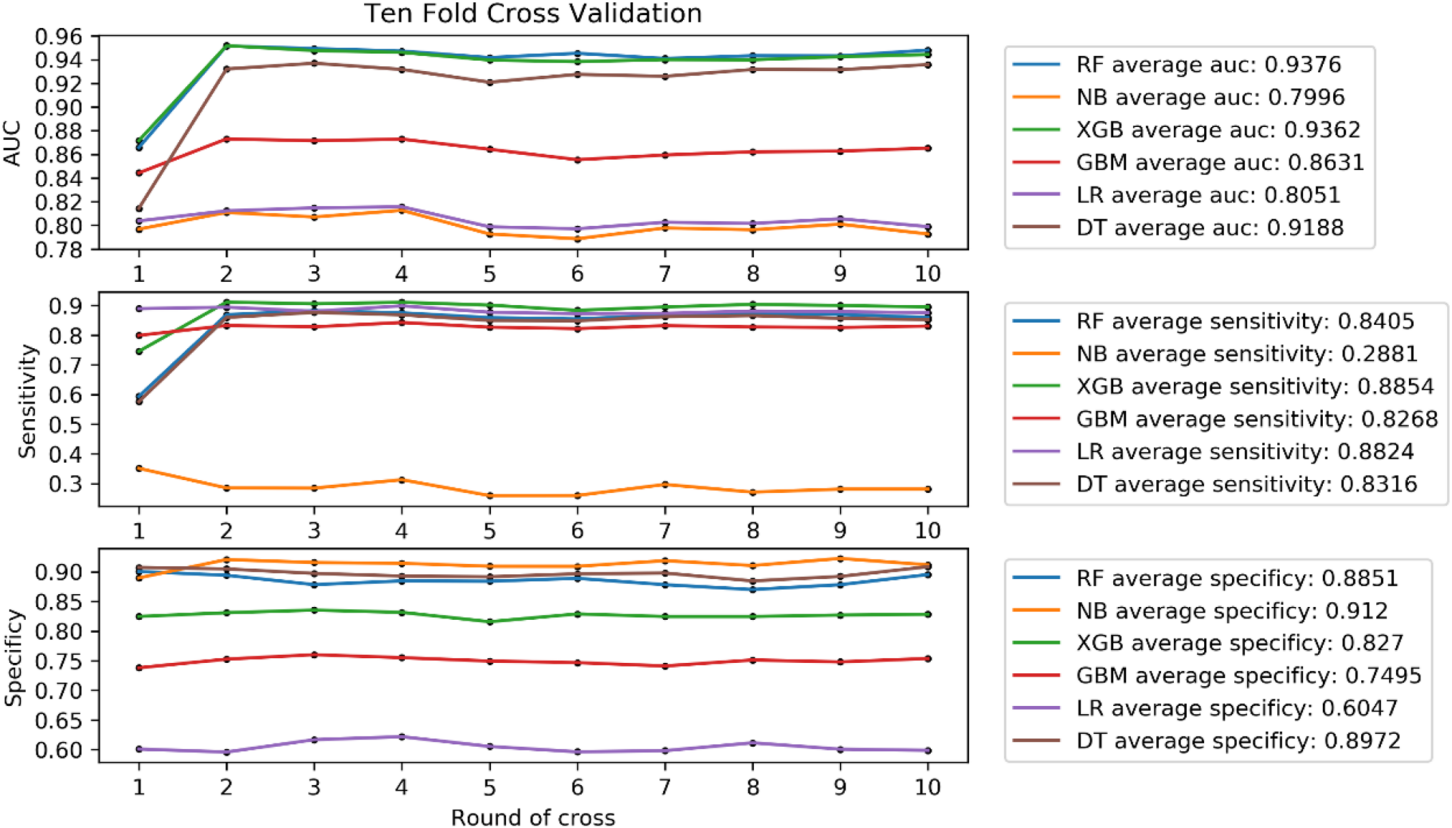
Tenfold cross-validation of 6 ML algorithms for predicting LNM in patients with PCa in the training set

**Fig. 3:**
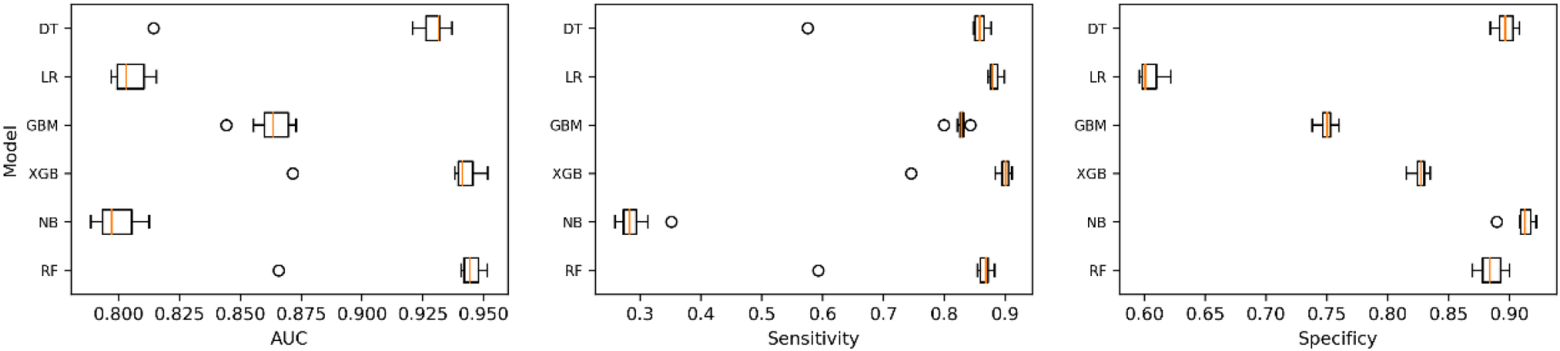
Prediction performance evaluation of training set prediction model

**Fig. 4:**
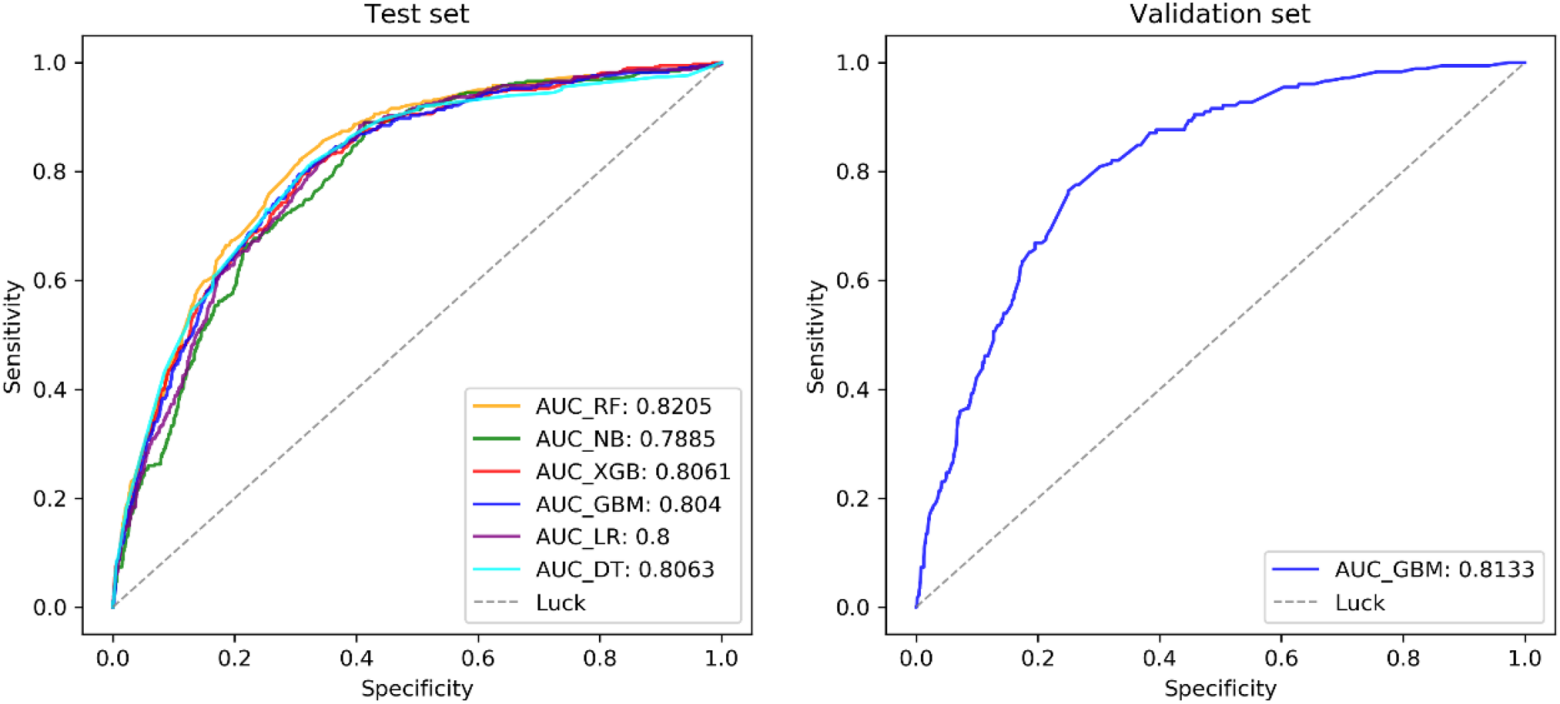
Receiver operating characteristic (ROC) curve of the test set and validation set prediction model

**Fig. 5:**
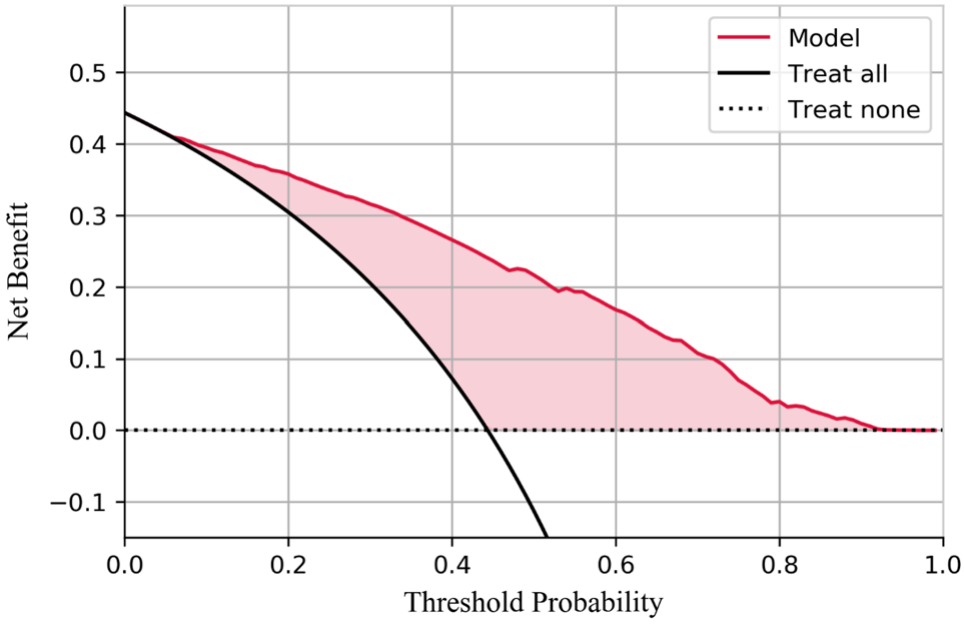
The decision curve analysis of the GBM model. In the figure, the red curve represents the predicted performance of the GBM model, respectively. In addition, there are two lines, which represent two extreme cases. The gray vertical line represents the hypothesis that all patients have LNM; the black horizontal line represents the hypothesis that no LNM occurs. The curve showed that when the LNM probability was between 0.1 and 0.9 in the training set. LNM could be discriminated when using this GBM predictive model to make clinical decisions

With lymph node status as a prognostic indicator, four factors (*p* < 0.05) in the above logistic regression analysis were determined to enter the model as variables. In the training set, ML algorithms including RF, NBC, XGB, GBM, LR and DT are executed to establish the prediction model. We used 10 times cross validation training for patients in the training group to adjust parameter balance and avoid over fitting of the model. The data set was divided into 10 parts, including 9 parts for training and 1 part for rotation test. The final accuracy rate averaged 10 times (Figs. 2–3). We found that RF model has the best prediction ability, AUROC = 0.82 (Fig. 4). AUROC of all models in the test set is > 0.7. F1 score value is suitable for evaluating the prediction performance of unbalanced samples. In the test set, GBM has the best prediction performance, significantly better than RF (F1 value: 0.838, sensitivity (recall): 0.877, specificity: 0.783; F1 value: 0.798, sensitivity (recall): 0.857, specificity: 0.709). Based on the aforementioned results, GBM was selected as the best prediction model for predicting LNM (Table 3). Furthermore, decision curve analysis (Fig. 5) shows the accuracy of GBM model.

**Table 3:** Performance of the developed models

Models	F1 score	Sensitivity (Recall)	Specificity
RF	0.798	0.857	0.709
NB	0.418	0.288	0.912
XGB	0.836	0.872	0.786
GBM	0.838	0.877	0.783
LR	0.775	0.882	0.605
DT	0.79	0.817	0.747

### Permutation feature of importance

**Fig. 6:**
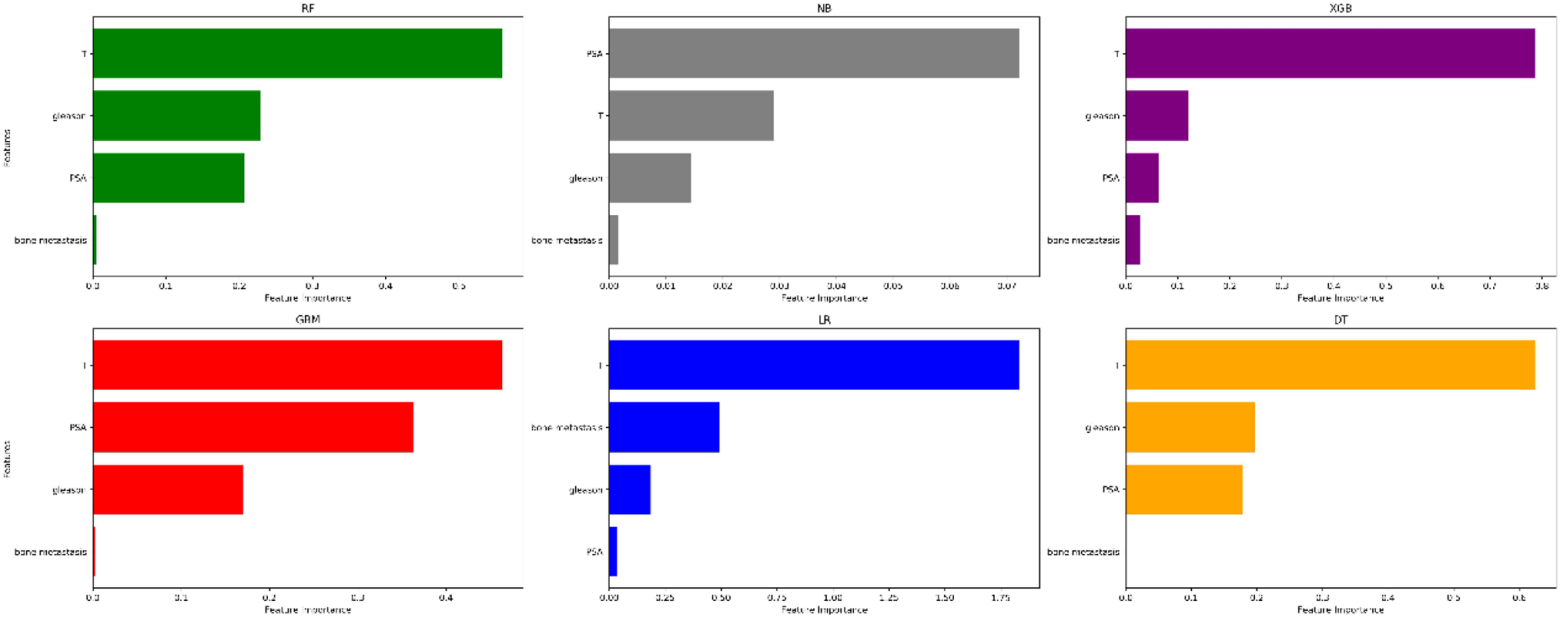
Relative importance ranking of each input variable for predicting models

In the six models, the relative importance order of each input variable is slightly different. T, PSA and Gleason are almost the first three indicators of each model, and bone metastasis is a lower indicator. (Fig. 6) In the GBM model, the order of relative importance of the variables from high to low is T, PSA, Gleason and bone metastasis.

### Calculator preliminary model

**Fig. 7:**
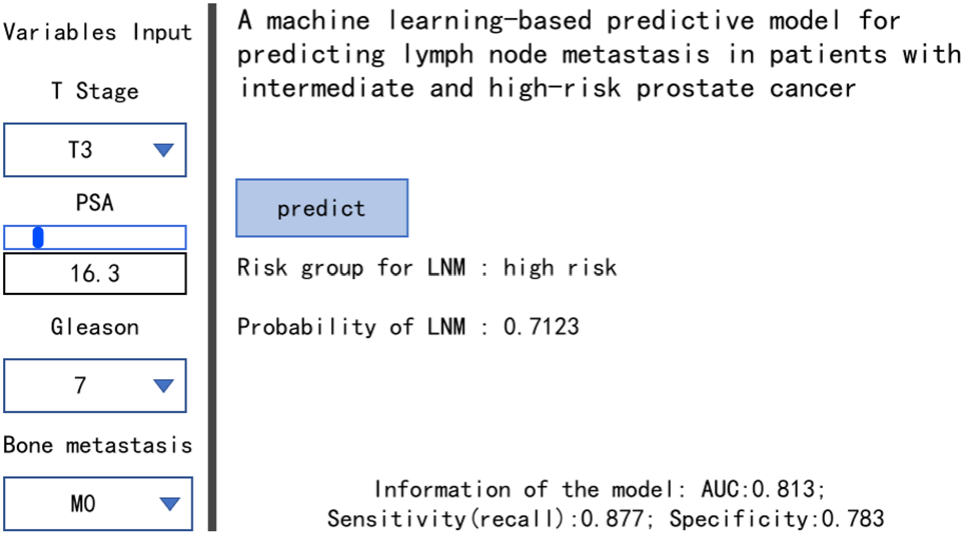
Calculator based on GBM model to predict LNM of intermediate- and high-risk PCa

The GBM model performs best among the six models. Accordingly, we have established a calculator preliminary model to promote the clinical application of this prediction model (Fig. 7).

## Discussion

LNM is a paramount prognostic factor for patients with PCa, and has been proved to be an important predictor of BCR survival, metastasis free survival and overall survival of PCa (Engel et al. 2010; Wilczak et al. 2018). Wessels et al. extracted prognostic information from the H&E histology of PCa and used the deep learning method to predict the LN status in PCa patients (Wessels et al. 2021). Hou et al. established PLNM risk calculator by integrating radiologist’s interpretation, clinicopathologic factors and MRIs, and using ML and deep migration learning algorithms (Hou et al. 2021). For the sake of accurately evaluating the risk of LNM, Some studies have designed different prediction models for lymph node prediction of intermediate- and high-risk PCa according to the detection pathway. Diamand R et al. reported and validated the LNM of patients treated with ePLND by nomogram, and provided a more reasonable cut-off value (Diamand et al. 2020). Ferraro DA et al. designed a new model by combining PSA, Gleason score and visual lymph node analysis on 68 Ga-PSMA-11 PET. Compared with the previously used clinical nomograms, this model has a remarkably improved the positive rate of LNM in the patient selecting to perform ePLND (Ferraro et al. 2020). In this study, we used the large sample size of SEER database and ML algorithm to develop six prediction models to predict LNM in the patients with intermediate- and high-risk PCa. Logistic regression analysis showed that T stage, Gleason score, PSA and bone metastasis were independent risk factors for pelvic LNM of intermediate- and high-risk PCa.

Among the six models, the AUC value of GBM model is the highest, and the prediction accuracy of other models for LNM is about 80%. RF model shows the best prediction performance before and after data balancing, with obvious advantages of high precision and fast speed; however, it also has the disadvantage of over fitting. F1 score, which represents the harmonic average of the accuracy rate and recall rate, is the final assessment parameter of the evaluating each model. According to the evaluation results of the test set, the prediction performance of GBM model is better than that of RF model. It can be seen that RF model may show over fitting in the training process, which makes it unsuitable for the data in the test set, while GBM model has the best prediction performance. To increase the application feasibility of this model, we developed a calculator to evaluate the individual probability of LNM in patients with intermediate- and high-risk PCa.

The results of this study showed that T stage, PSA, Gleason score and bone metastasis were the most important predictors in the patients with intermediate- and high-risk PCa. As an important indicator of tumor progression, T stage is positively correlated with LNM in a large number of tumors (Barriera-Silvestrini et al. 2021). A large number of research data in this study show that the level of high PSA will increase the rate of lymph node invasion, which is contrary to the results of the previous studies. The possible reason is PSA may be more meaningful in D'Amico risk stratification. The increase of Gleason score also increases the risk of lymph node invasion (Turk et al. 2018). Bone metastasis is significantly related to LNM of PCa, which can provide some ideas for follow-up research, that is, consider the existence of metastasis of other sites as a factor before patients have LNM.

The EAU guidelines used Briganti’s nomogram prediction model to screen ePLND patients. The advantage of this study is to compare several models head-to-head with the nomogram model. The sensitivity, specificity and AUC of the nomogram are 0.882, 0.705 and 0.80, respectively, while the sensitivity, specificity and AUC of GBM are 0.877, 0.783 and 0.813, respectively. It shows that GBM in the six predictive models has the best predictive value for LNM in the patients with intermediate- and high-risk PCa. To further facilitate clinical application, we designed a preliminary calculator model that can quickly calculate the probability of LNM.

Of course, this study has several limitations. First, this study is a retrospective study, which may have some selection bias. Second, SEER database lacks more data such as tumor volume, percentage of positive tissue cores, testosterone level, and so on. In addition, the external validation set data is small, and more sample sizes need to be included to test the effectiveness of the model. Finally, although we have corrected the sample imbalance problem of SEER dataset as much as possible, this problem will still interfere with the results and affect the generalization ability of the model.

## Conclusion

This research has developed and validated six prediction models using ML algorithm, of which GBM model has the best performance. Based on this algorithm, a preliminary model of the calculator is designed, and then the local LNM probability in patients with intermediate- and high-risk PCa can be individually predicted according to the existing clinical characteristics, which can help clinicians quickly and accurately assess the risk of LNM, finally, precise therapy.

## Data Availability

The data on which the study is based is available from the repository and can be downloaded at the following link (https://seer.cancer.gov). Relevant information will be provided upon reasonable request.

## Abbreviations

LNM: Lymph node metastasis
PCa: Prostate cancer
ML: Machine learning
RF: Random forest
NBC: Naive Bayesian classifier
XGB: Xgboost
GBM: Gradient boosting machine
LR: Logistic regression
DT: Decision tree
ROC: Receiver operating characteristic
AUC: Area under curve
PLNM: Pelvic lymph node metastasis
ePLND: Extended pelvic lymph node dissection
SEER: Surveillance, epidemiology and end results
BCR: Biochemical recurrence
